# Punctured Wound of Heart

**Published:** 1886-01-20

**Authors:** Chas. Chassaignac

**Affiliations:** Louisiana


### PUNCTURED WOUND OF HEART.

BY CHAS. CHASSAIGNAC, M.D., OF LOUISIANA.

The very able paper by Dr. A. B. Miles upon gunshot wounds of the heart, published in your August number, recalls a case which came under my observation shortly before in the Charity Hospital. The case is one of punctured wound of the heart. Though the injury to that organ is slight compared to most of those cited in the article, it tends to prove the correctness of Dr. Miles' deductions about the shock and the length of time an individul can live after a wound of the organ in question. As the observation may have one one or two other points of interest, I feel warranted in transmitting it to you concisely.

John Brook, colored, aged 39, was brought to Ward 3, on July 12th, having received a punctured wound of right side of chest. The blade had entered between the second and third ribs, producing a cut one-half inch long, cutting through junction of third rib with its cartilage, then ranging downwards and inwards, penetrating the lung. as evidenced by profuse hemorrhage, escape of air, and coughing up of blood. *The shock was slight*, his pulse being full and beating seventy-two to the minute, temperature normal. Next day, July 13th, his *pulse was* 64 to the minute, temperature 100° ; there was a slight recurrence of hemorrhage, chiefly venous, upon changing the dressing ; he took a little nourishment. July 15th, little change, but takes less nourishment and is delirious at intervals. July 16h, seems stronger, more rational; and nourishes well. July 17th, temperature high, going to nearly 105° in the evening, pulse feeble and now rapid, respiration heavy. July 18th, in the afternoon, suddenly grew faint and died by syncope *six days* after reception of the injury.

Post-mortem examination showed that the weapon after going through the lung had penetrated the pericardium and inflicted a wound about three-fourths of an inch long and one-eighth of an inch deep in the wall of the right ventricle. The edges of this cut were granulating and seemed already to have slightly adhered. In addition, the pleural cavity contained a large quantity of blood, the lung was carnified, and the pericardium held nearly an ounce of pus. For the account of the autopsy, I am indebted to Mr. Charbonnet, interne of the ward, who witnessed the coroner's examination.

You will permit me to make a query. Could not the remarkable slowness of the pulse at the onset, notwithstanding the gravity of the wound and the amount of hemorrhage, have been due to irritation of the pneumogastric nerve through the filaments which are disturbed both on the surface and in the substance of the injured ventricle ?—*N. O. Med. & Surg. World.*

